# The Multiple Effects of RE Element Addition in Non-Oriented Silicon Steel

**DOI:** 10.3390/ma18020401

**Published:** 2025-01-16

**Authors:** Guobao Li, Yongjie Yang, Zhenghua He, Yuhui Sha

**Affiliations:** 1 Baoshan Iron & Steel Cooperation Limited, Shanghai 201999, China; yangyongjie@baosteel.com; 2 Key Laboratory for Anisotropy and Texture of Materials (Ministry of Education), Northeastern University, Shenyang 110819, China; hezhenghua@mail.neu.edu.cn; 3 School of Materials Science and Engineering, Shenyang University of Technology, Shenyang 110870, China

**Keywords:** silicon steel, rare earth element, recrystallization texture, inclusion, grain size

## Abstract

High-grade non-oriented silicon steel with high magnetic induction and low iron loss produced with low carbon emissions is crucial for the development of new energy and energy-saving motors. In this paper, the trace mixed rare earth (RE) elements exhibit a great potential to enhance magnetic properties in a lower carbon emission process by multiple effects on microstructure, texture, and inclusion in non-oriented silicon steel. With the trace-doped RE elements (0.004–0.030%), RE-rich precipitates preferentially form and subsequently adsorb fine inclusions below 1 μm to transform into spherical or ellipsoidal shape, which results in a significant increase in final recrystallization grain size. Moreover, the favorable λ texture (<001>//ND) is promoted while the detrimental γ texture (<111>//ND) is reduced, owing to the advantages in size and quantity of λ grains during the nucleation process. The improved magnetic properties of higher B50 and lower P15/50 are achieved with 0.004% RE at lower annealing temperature ranges. The increased λ texture is attributed to the heterogeneity in microstructure and texture as well as the grain boundary segregation of RE elements. However, a higher RE content (0.072%) leads to a deterioration in magnetic performance due to the formation of more stable RE-rich precipitates, smaller grains, and stronger γ texture. An iron loss calculation model was also proposed to guide the design of high-grade non-oriented silicon steel by incorporating the multiple effects of RE elements on grain size, recrystallization texture, and inclusion.

## 1. Introduction

High-grade non-oriented silicon steel is an essential material for new energy motors due to low iron loss, high magnetic permeability, and excellent mechanical properties. Extensive research has been dedicated to optimizing grain size and increasing favorable textures with easy magnetization direction <100> in the rolling plane as two critical factors for magnetic properties. Grain growth is seriously inhibited by a variety of inclusions distributed throughout silicon steel sheets, including MnS, Cu_2_S, TiN, TiC, and Al_2_O_3_, so that higher annealing temperature and special rolling process are required to promote grain growth [1,2,3,4]. The λ (<100>//ND) columnar grains are attained by solidification and vacuum annealing [5,6,7]. Strong λ and Goss ({110}<001>) textures are produced in annealed silicon steel sheets via strip casting [8,9,10], critical rolling [11], asymmetric rolling [12,13,14,15], as well as the modified rolling parameters and annealing atmospheres [16,17,18,19]. Nano- and submicron-sized inclusions can also lead to the deterioration of magnetic properties by impeding magnetic domain wall movement and grain growth [20,21].

Rare earth (RE) elements have a strong affinity for oxygen (O) and sulfur (S) and inhibit the formation of MnS, Cu_2_S, and Al_2_O_3_ particles [22,23,24,25,26,27,28]. An appropriate RE element addition can effectively coarsen inclusions and reduce fine inclusions [22,23,24,25,26,27,28,29,30]. Additionally, RE elements have been reported to promote beneficial texture development in silicon steel. Strong Goss texture is achieved with 0.003% Ce by enhanced hot rolling and normalizing texture [30]. Goss and cube ({100}<001>) textures increase along with decreased γ texture at 0.0051% Ce due to the reduction in inclusions and cold rolling γ texture [31]. The improved magnetic property with 0.0066% La is associated with strong {110}<110> and weak {112}<110> textures [32,33]. He et al. [34] found that strong λ and {113}<361> textures are obtained with RE element addition from 0.005% to 0.045% by the inhibited nucleation and growth of γ grains. Ren et al. [35,36] reported that the addition of 0.0006% La promoted the magnetic properties of non-oriented silicon steel by reducing the number of MnS particles and strengthening the {100}<021> texture, but the magnetic properties deteriorated after adding 0.0006% La and 0.007% Ce due to the increase in RE sulfides and deterioration of the grain size and textures. Wang et al. [37] reported that the magnetic properties of W350 non-oriented silicon steel were enhanced by adding 0.0025% La-Ce alloy due to the coarsening of inclusions. Qin et al. [38,39] reported that 0.012% Y promoted the η texture due to an increased occurrence of shear bands, as well as enhanced {001}<130> and {114}<481> texture resulting from a greater number of nucleation sites and the high mobility of grain boundaries in 6.5 Si steel. Therefore, trace RE elements can mitigate harmful inclusions to increase grain size and favorable texture, which is expected to improve magnetic properties and simplify the final annealing process. RE elements have great potential to reduce carbon emissions during the manufacturing and service of high-grade non-oriented silicon steel.

Mixed RE elements composed of La, Ce, Pr, and Nd are more cost-saving for steelmaking; however, the effects of mixed RE elements in non-oriented silicon steels are unclear yet. In this paper, the multiple impacts of mixed RE elements on grain size, recrystallization texture, inclusion, and magnetic properties were systematically investigated with mixed RE elements of 0.004~0.072%. A calculation model for iron loss was further developed based on iron loss separation by incorporating grain size, texture, and inclusion characteristics.

## 2. Materials and Methods

Silicon steel ingots were prepared in a vacuum induction furnace with an RE mixture including Ce, La, Nd, and Pr. The chemical compositions are presented in Table 1. No RE elements were added in Sample 1, while the total RE content in other samples was 0.004%, 0.030%, and 0.072%, respectively. The ingots were forged into slabs with a thickness of 30 mm at 1100 °C, then reheated at 1150 °C for 2 h and hot-rolled to a thickness of 2.1 mm with a finishing rolling temperature of 850 °C. These hot-rolled bands were normalized at 1000~1050 °C for 2~5 min, subsequently cold-rolled to 0.50 mm, and finally annealed between 800 and 1100 °C in nitrogen atmosphere. To investigate the effect of RE addition on grain growth, silicon steel sheets with varying RE contents were annealed at 850 °C, 900 °C, 950 °C, 1000 °C, 1050 °C and 1100 °C for 10 min. To compare the effects of RE elements on the recrystallization texture, silicon steel sheets with uniform grain sizes (30 μm, 50 μm, 80 μm, 100 μm, 140 μm, and 190 μm) were selected for varying RE element contents.

The macro-texture was quantitatively examined on different thickness layers using an X-ray diffractometer (X’Pert PRO, Almelo, The Netherlands) with Co *K*_α1_ radiation at 35 kV and 40 mA. Here, the thickness layer is defined by the parameter *S* = 2*a*/*d*, where *a* represents the distance from the center layer and *d* is sheet thickness. Orientation distribution functions (ODFs) were calculated based on three incomplete pole figures of {110}, {200}, and {211}. The micro-texture was investigated on longitudinal sections defined by rolling direction (RD) and normal direction (ND) using a field emission gun scanning electron microscope (JEOL 6500F, JEOL, Tokyo, Japan) equipped with an electron backscatter diffraction (EBSD) acquisition system. A texture component was determined with a tolerance angle of 15°. The segregation of RE element was analyzed on the intergranular fracture surface of recrystallized samples using X-ray photoelectron spectroscopy (XPS) conducted with the Model Axis Supra instrument (Shimadzu Kratos Analytical, Manchester, UK). Two regions for each sample were performed by XPS characterization to ensure the accuracy of the data results. The magnetic flux density at 5000 A/m (B50) and the iron loss at 1.5 T and 50 Hz (P_15/50_) were measured using an IWATSU B-H analyzer (SY-8232, IWATSU, Tokyo, Japan).

## 3. Results

### 3.1. Microstructure and Texture After Normalized Annealing, Cold Rolling, and Recrystallization

**Figure 1:**
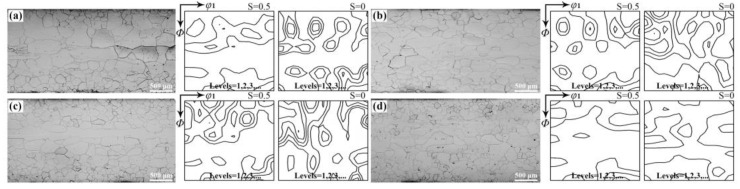
Microstructure and *φ*_2_ = 45° sections of ODFs of normalized bands with different RE contents: (**a**) 0, (**b**) 0.004%, (**c**) 0.030%, (**d**) 0.0720%.

Figure 1 illustrates the microstructure and texture of normalized hot bands with different RE contents. The normalized hot bands are composed of fully recrystallized grains, where a strong Goss texture is located in the sub-surface layer, while λ and γ textures are predominantly found at the central layer. A notable characteristic of the texture in these normalized hot bands is that the λ textures are strongest when the RE element content is below 0.004%, whereas the γ texture is enhanced when the RE content exceeds 0.072%.

**Figure 2:**
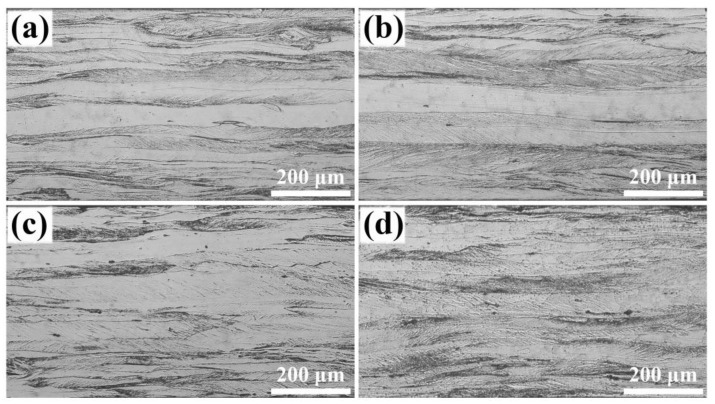
Microstructure of cold-rolled silicon steel sheets with different RE contents: (**a**) 0, (**b**) 0.004%, (**c**) 0.030%, (**d**) 0.0720%.

Figure 2 shows the cold-rolling microstructure with different RE contents. In the sub-surface layer, deformed grains contain a large number of in-grain shear bands inclined by 20~30° to the rolling direction. Conversely, deformed grains in the central layer exhibit a larger size and reduced shear bands. However, when RE content exceeds 0.030%, the deformed grains in the central layer diminish while shear bands gradually increase.

**Figure 3:**
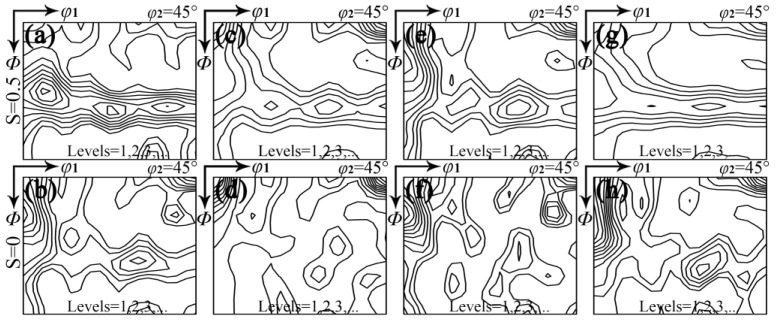
Constant *φ*_2_ = 45° sections of ODFs in cold-rolled silicon steel sheets with different RE contents of (**a**,**b**) 0, (**c**,**d**) 0.004%, (**e**,**f**) 0.030%, (**g**,**h**) 0.0720% on (**a**,**c**,**e**,**g**) sub-surface layer and (**b**,**d**,**f**,**h**) central layer.

As illustrated in Figure 3, the cold-rolling texture with different RE contents consists primarily of γ-fiber and α-fiber (<110>//RD), and there is a notable difference between sub-surface and central layers. In the sub-surface layer, the intensity of the γ texture is greater than the α texture. A significant observation in the central layer is that the γ texture initially weakens at 0.004–0.030% RE and then strengthens as RE content increases. Based on the cold rolling microstructure, it can be inferred that the wider deformed grains in the central layer are mainly the α texture, whereas the narrower deformed grains containing shear bands belong to the γ texture [15,40,41,42,43,44,45]. The cold-rolling microstructure and texture associated with RE content ultimately influence the grain size and texture after final annealing.

**Figure 4:**
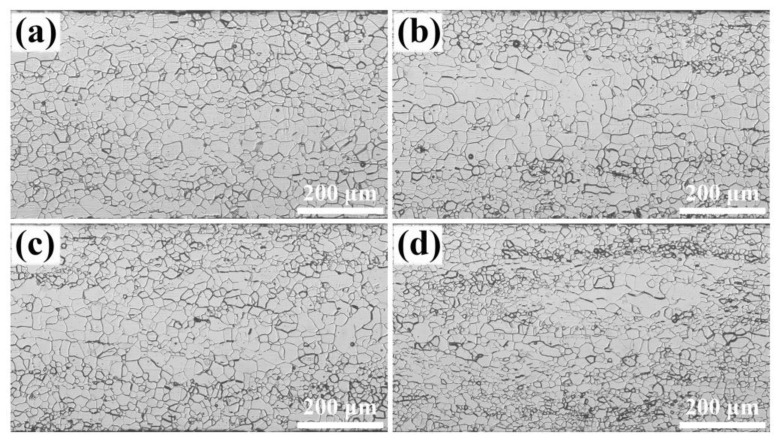
Microstructure of completely recrystallized silicon steel sheets with different RE contents: (**a**) 0, (**b**) 0.004%, (**c**) 0.030%, (**d**) 0.0720%.

Figure 4 displays the microstructure in silicon steel sheets that just completed primary recrystallization after annealing at 800 °C for 5 min. An evident heterogeneity in grain size can be observed: recrystallized grains in the central layer exhibit a relatively larger size compared to the sub-surface layer. Without the RE element, the average grain size increases from 27 μm to 34 μm with the decreasing thickness parameter *S* (Figure S1), which is attributed to the fewer nucleation sites in the central layer due to the lower stored energy of deformed α grains. At 0.004%~0.03% RE, the grain size significantly increases because of a greater number of wider α deformed grains distributed in the central layer. However, when RE content reaches 0.072%, more γ deformed grains with higher stored energy, along with narrower α deformed grains, create a substantial number of nucleation sites along grain boundaries, which results in a significant reduction in grain size throughout sheet thickness after primary recrystallization.

**Figure 5:**
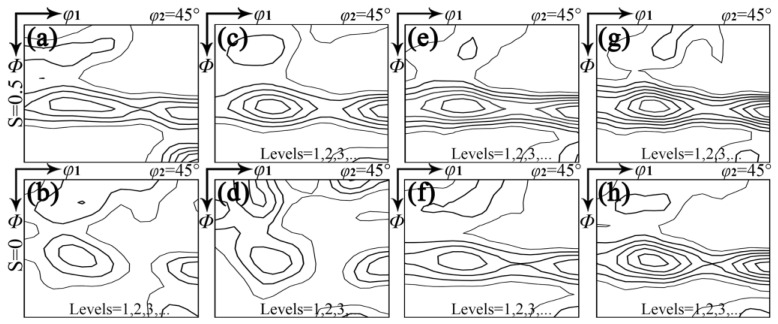
Constant *φ*_2_ = 45° sections of ODFs in completely recrystallized silicon steel sheets with different RE contents of (**a**,**b**) 0, (**c**,**d**) 0.004%, (**e**,**f**) 0.030%, (**g**,**h**) 0.0720% on (**a**,**c**,**e**,**g**) sub-surface layer and (**b**,**d**,**f**,**h**) central layer.

Figure 5 gives the texture of silicon steel sheets annealed at 800 °C for 5 min with different RE contents. The recrystallization texture consists mainly of γ-fiber with a peak at {111}<112>, λ-fiber with peaks at cube and {001}<210>, and Goss. A weaker γ texture and stronger λ texture form in the central layer compared to the sub-surface layer. As RE content increases, the recrystallization texture in the sub-surface layer has no obvious change in addition to a slight increase of {111}<112>. Conversely, λ-fiber initially strengthens and then weakens, while γ-fiber has just the opposite change with increasing RE content in the central layer. The most pronounced increase in λ-fiber is achieved at 0.004% RE, which is mainly attributed to the enhanced α-fiber and broader α deformed grains in the central layer [34].

**Figure 6:**
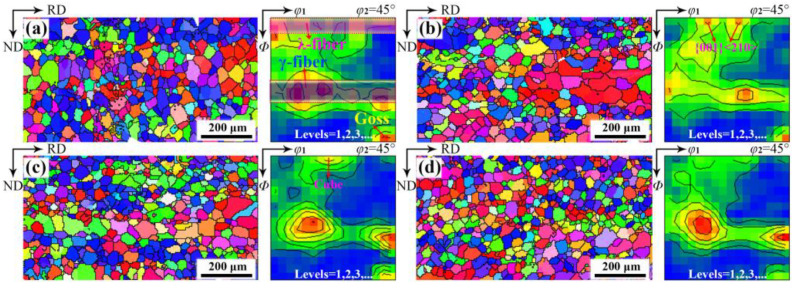
Orientation image maps and constant *φ*_2_ = 45° sections of ODFs in silicon steel sheets annealed at 800 °C for 10 min with different RE contents: (**a**) 0, (**b**) 0.004%, (**c**) 0.030%, (**d**) 0.072%.

After prolonged annealing at 800 °C for 10 min, the grain size and texture are similar to just-complete recrystallization. As shown in Figure 6, the microstructure heterogeneity retains throughout the sheet thickness, and the grain size initially increases and then decreases with increasing RE content. The recrystallization texture comprises {111}<112>, Goss, and λ-fiber with intensities dependent on RE content. It is thus confirmed that an appropriate RE addition can optimize recrystallization grain size and texture by regulating the microstructure and texture of cold-rolled sheets.

### 3.2. Inclusion After Primary Recrystallization

**Figure 7:**
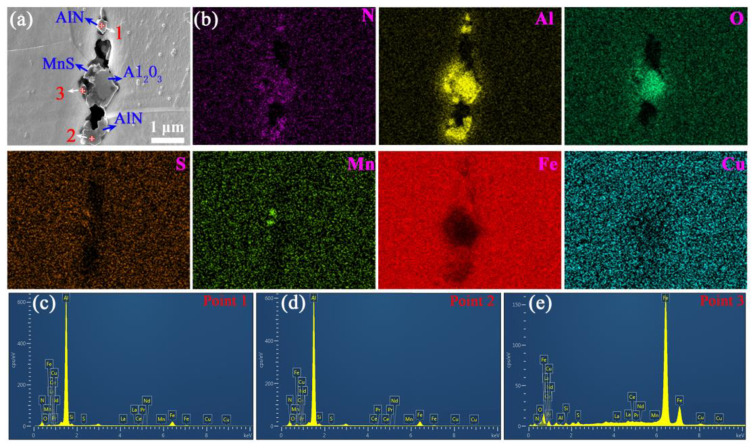
(**a**) SEM micrograph for various types of inclusions, (**b**) EDS at local mapping, (**c**) point 1, (**d**) point 2, and (**e**) point 3 among the selected points of silicon steel sheet without RE elements.

Various inclusions, such as MnS, Cu_2_S, AlN, and Al_2_O_3_, are widely distributed in silicon steel sheets without RE elements after primary recrystallization (Figure 7). These particles can exist in either individual or composite types with sizes from 0.2 to 1 μm and a few exceeding 1 μm.

**Figure 8:**
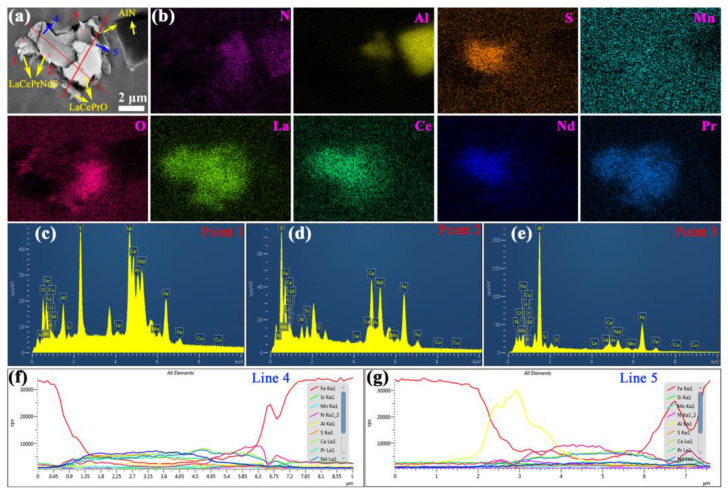
(**a**) SEM micrograph for irregular inclusion, (**b**) EDS at local mapping, (**c**) point 1, (**d**) point 2, and (**e**) point 3 among the selected points, as well as (**f**) line 4 and (**g**) line 5 of selected lines of silicon steel sheet with 0.004% RE.

**Figure 9:**
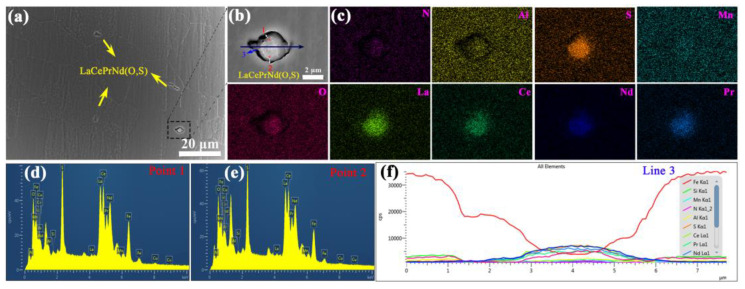
(**a**) SEM micrograph and (**b**) local enlarge for irregular inclusion, (**c**) EDS at local mapping, (**d**) point 1 and (**e**) point 2 of selected points, as well as (**f**) selected line 3 of silicon steel sheet with 0.004% RE.

**Figure 10:**
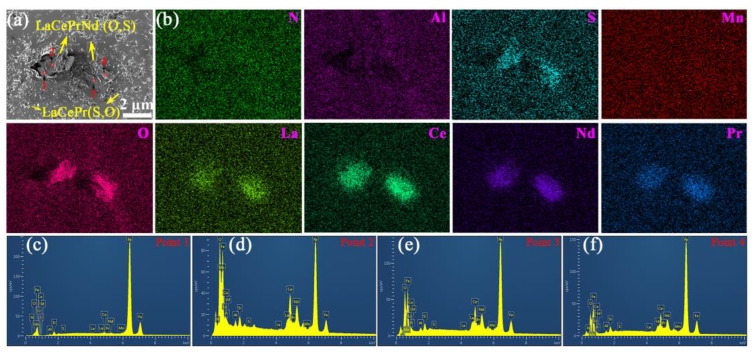
(**a**) SEM micrograph, (**b**) EDS at local mapping, (**c**) point 1, (**d**) point 2, (**e**) point 3, and (**f**) point 4 among the selected points selected points of silicon steel sheet with 0.072% RE.

The particles below 1 μm are significantly reduced, while composite particles exceeding 1 μm appear in silicon steel sheets containing 0.004% RE. These composite particles exhibit irregular shape and consist of multiple elements including Ce, La, Pr, Nd, Mn, Al, N, O, and S elements (Figure 8). The larger particles are formed from rare earth oxygen sulfide core and adsorption of MnS, AlN, and Al_2_O_3_ fine particles. Many composite particles display spherical or ellipsoidal shapes with sizes exceeding 5 μm (Figure 9). Compared to the addition of an individual RE element [30,31,32,33], larger and fewer inclusions can be achieved by a lower content of mixed RE elements. In contrast, RE element-rich sulfides and oxides, ranging from 0.5 to 1 μm, are widely distributed in silicon steel sheets containing 0.072% RE (Figure 10). This indicates that RE elements preferentially form sulfides and oxides due to their strong affinity for oxygen and sulfur. An appropriate RE content of 0.004% to 0.030% promotes the adsorption of MnS, AlN, and Al_2_O_3_ fine particles and mitigates harmful inclusions. However, the excessive addition of RE results in numerous small-sized RE-rich precipitates.

**Figure 11:**
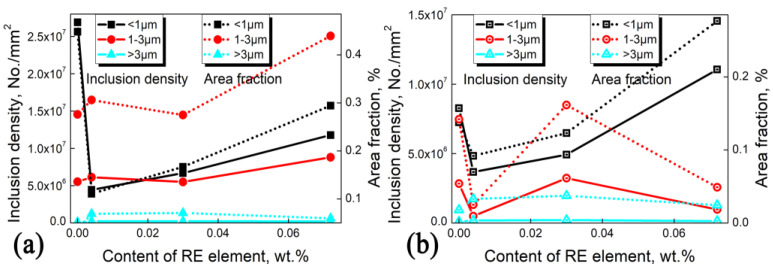
Inclusion density with different RE contents after annealing at (**a**) 850 °C and (**b**) 1000 °C. The solid and dashed curves indicate the number density and area fraction of inclusions, respectively.

Figure 11 shows the density and size distribution of inclusions in silicon steel with different RE contents. Fine inclusions below 1 μm are predominant without RE addition and significantly decrease by 86% with 0.004% RE addition. However, at 0.072% RE, the density of inclusions below 1 μm reaches as much as 1.1 × 10^7^/mm^3^. As the annealing temperature is raised to 1000 °C, the quantity of fine inclusions decreases notably without RE addition, whereas it does not exhibit a significant change with RE addition. This demonstrates that the trace addition of RE elements can effectively reduce fine inclusions in silicon steel, and a high annealing temperature does not substantially coarsen the RE-rich precipitates with higher thermal stability.

### 3.3. Microstructure and Texture During Grain Growth

**Figure 12:**
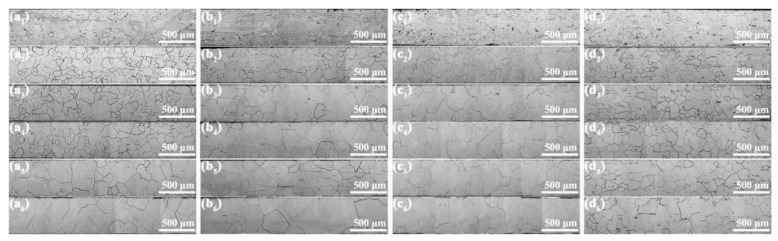
Microstructure of silicon steel sheets with RE contents of (**a**) 0, (**b**) 0.004%, (**c**) 0.030%, (**d**) 0.0720% annealed at (**1**) 850 °C, (**2**) 900 °C, (**3**) 950 °C, (**4**) 1000 °C, (**5**) 1050 °C, (**6**) 1100 °C, respectively.

**Figure 13:**
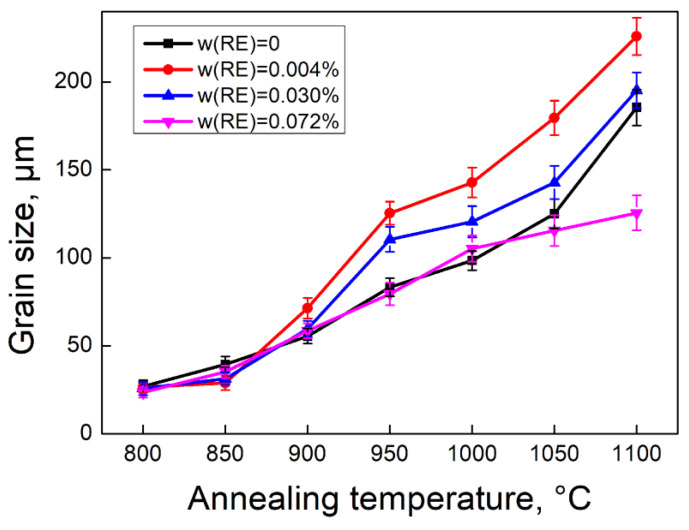
Variation in average grain size with annealing temperatures in silicon steel sheets with different RE contents.

An optimized grain size is essential for improving magnetic properties. To investigate the effect of RE addition on grain growth, silicon steel sheets with varying RE contents were annealed at 850 °C, 900 °C, 950 °C, 1000 °C, 1050 °C, and 1100 °C for 10 min, as shown in Figure 12. Figure 13 further summarizes the relationship between average grain size and annealing temperature. Without RE addition, the recrystallized grains grow gradually below 950 °C, and rapidly above 1000 °C due to the reduction in pinning force. In contrast, the rapid grain growth occurs with the temperature exceeding 850 °C with RE content ranging from 0.004% to 0.03%. Therefore, coarse grains can be obtained at lower annealing temperatures with trace amounts of RE addition. However, the grain size shows little increase at annealing temperatures above 1000 °C with 0.072% RE addition due to the higher density of stable RE-rich particles. This phenomenon is different from the effect of an individual RE element on the grain size in silicon steel, where a high content of RE element [30] or a small range of RE element is required for large recrystallization grains [31,32,33]. The mixed RE elements exhibit a more significant purification effect on inclusions due to their strong adsorption and good stability.

**Figure 14:**
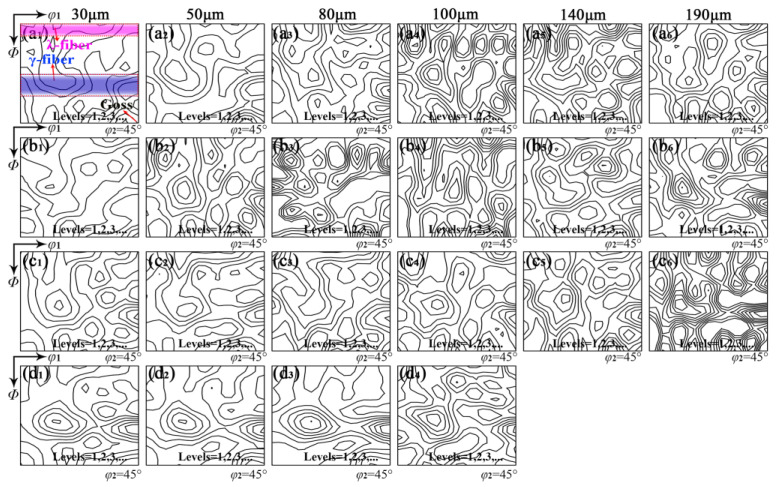
Constant *φ*_2_ = 45° sections of ODFs in silicon steel sheets with RE contents of (**a_1_**–**a_6_**) 0, (**b_1_**–**b_6_**) 0.004%, (**c_1_**–**c_6_**) 0.030%, (**d_1_**–**b_4_**) 0.0720% for the average grain size of 30 μm (1), 50 μm (2), 80 μm (3),100 μm (4), 140 μm (5), 190 μm (6).

Both magnetic induction and iron loss of non-oriented silicon steel depend on recrystallization texture. Silicon steel sheets with a grain size between 30 μm and 190 μm were selected to compare the RE effect on recrystallization texture, where the texture in the central layer was used to represent the overall texture based on measurement from XRD (Figure 5) and EBSD (Figure 6). Figure 14 demonstrates the texture characteristic of silicon steel with different RE contents in different grain sizes. Without RE elements, the texture remains relatively unchanged below 80 μm, while λ fiber tends to strengthen in contrast to Goss and γ fiber above 100 μm. At 0.004–0.030% RE, γ-fiber and Goss gradually decrease while λ fiber strengthens with increasing grain size below 80 μm. Conversely, λ fiber diminishes and γ fiber increases above 140 μm. This phenomenon is mainly attributed to the size advantage of λ grains over γ and Goss grains in the central layer during primary recrystallization [16,17,44,46]. With the addition of 0.072% RE, γ fiber remains dominant and consistent during grain growth below 140 μm.

### 3.4. Magnetic Property During Grain Growth

**Figure 15:**
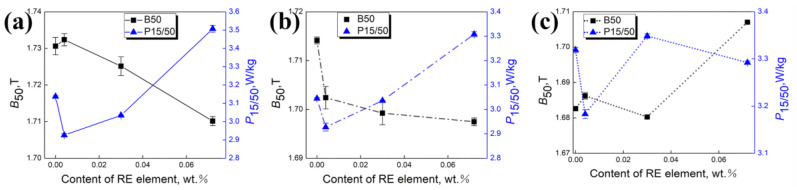
Magnetic induction (B_50_) and iron loss (P_15/50_) of silicon steel sheets with different RE contents when annealing at (**a**) 900 °C, (**b**) 1000 °C, and (**c**) 1100 °C.

Figure 15 compares magnetic induction (B_50_) and iron loss (P_15/50_) at various annealing temperatures. When annealing at 900 °C, magnetic induction initially rises and then declines with increasing RE content, just opposite to iron loss. The optimal property is achieved with 0.004% RE. However, magnetic induction and iron loss exhibit a complex relationship with RE content above 950 °C. This is primarily due to complex alterations in grain size and texture dependent on RE content at higher annealing temperatures.

**Figure 16:**
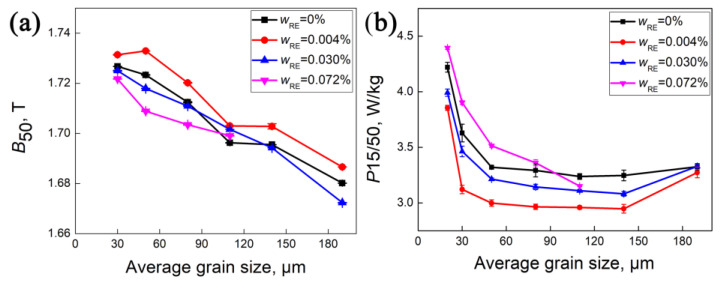
Relationship of magnetic induction (**a**) (B_50_) and iron loss (**b**) (P_15/50_) with average grain size of silicon steel sheets for different RE elements.

Figure 16 shows the variation in magnetic properties with grain size for different additions of RE elements. The magnetic induction (B_50_) gradually decreases with increasing grain size except for 0.004% RE. The silicon steel sheet with 0.004% RE has the highest B_50_ within the whole grain size range. The iron loss (P_15/50_) exhibits a sharp decrease in the small grain size range, and then a plateau followed by a rise in the large grain size range, with a minimum value at 120–150 μm. Generally, P_15/50_ is the lowest at 0.004% RE, but highest at 0.072% RE.

Since the effect of grain size on magnetic properties has been excluded, the observed differences in magnetic properties can be attributed to texture and inclusions. It can be inferred that the increase in B_50_ with 0.004% RE is due to the enhanced λ fiber. The reduction in P_15/50_ is contingent upon the favorable texture and a lower number of inclusions. The marked difference in P_15/50_ for grain sizes less than 140 μm is related to the texture and inclusions, while the diminished difference near 190 μm is attributed to the reduction in inclusions without RE addition and the increased γ fiber with RE addition.

## 4. Discussion

### 4.1. Multiple Effects of RE Elements on Microstructure and Texture

The trace RE elements (0.004–0.030%) can preferentially form RE-rich sulfides and oxides, then adsorb fine MnS, Al_2_O_3_, and AlN to transform into spherical or ellipsoidal shaped and larger composite particles. More large-sized inclusions are achieved by mixed RE elements with a lower addition than individual RE elements [24,25]. The reduced density and coarsening of inclusions by trace-doping of mixed RE elements (0.004%) can significantly decrease the pinning force on grain growth, leading to a larger grain size at lower annealing temperatures without a special rolling or annealing process [8,13,14,15].

The 0.004–0.030% RE addition can enhance the heterogeneity of the microstructure and texture in cold-rolled sheets. The resultant advantages in grain size and quantity for λ fiber during nucleation promote the preferred growth of λ grains over γ grains during the grain growth stage. In contrast, the higher content of 0.072% RE tends to benefit the nucleation and grain growth of γ grains, which is attributed to the increased γ deformed texture and the retarded grain growth.

Without RE addition, recrystallization texture does not change significantly in grain size range from 20 μm to 140 μm due to the uniformly decreased inhibiting force on grain growth for various texture components with rising annealing temperatures. The λ texture in the central layer is preserved during high-temperature annealing by the advantages in grain size and number established during nucleation. In the case of 0.004–0.030% RE, the enhanced λ fiber and weakened γ fiber form below 1000 °C, contrary to the annealing temperature above 1000 °C. Texture development above 1000 °C is not only attributed to the change in grain size and number but also the reduced density of particles by trace RE elements.

**Figure 17:**
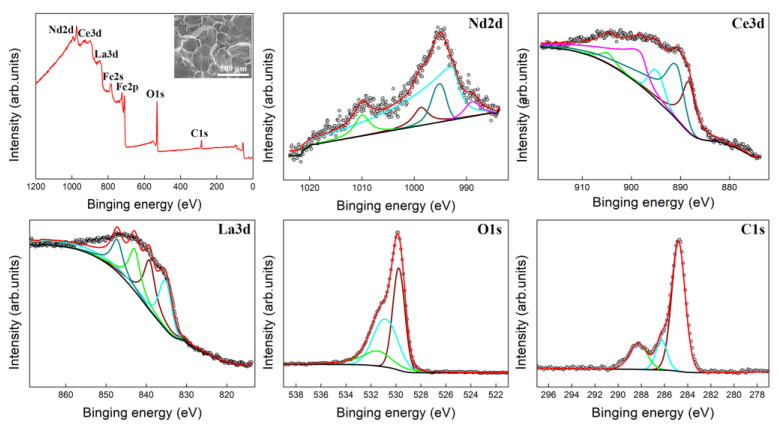
XPS of fracture surface of silicon steel sheets with 0.004% RE annealed at 1000 °C for 10 min and impacted in liquid nitrogen.

The grain boundary segregation of trace RE elements has a beneficial effect on recrystallization texture [34,47,48]. To investigate the grain boundary segregation, the silicon steel sheet was impacted in liquid nitrogen for intergranular fracture surface analysis. Figure 17 displays the XPS of the fracture surface of silicon steel sheets with 0.004% RE annealed at 1000 °C for 5 min and impacted in liquid nitrogen. The fracture morphology at liquid nitrogen temperature presents a typical transgranular cleavage fracture. Therefore, the distribution of trace RE elements at the grain boundaries can be analyzed using XPS to illustrate the segregation of RE elements in the grain boundary. Small peaks of La, Ce, and Nd elements indicate the trace Nd, Ce, and La elements on the fracture surface. The trace Nd, Ce, and La elements on the fracture surface confirm the RE segregation at grain boundaries. However, the segregation diminishes when the annealing temperature exceeds 1000 °C. This suggests that the earlier nucleation and growth are retarded by grain boundary segregation of RE elements at 1000 °C, and λ grains are less affected by larger grain size than γ grains. Above 1000 °C, either the advantages for λ grains in grain size and number over γ grains or grain boundary segregation of RE elements decrease rapidly. Therefore, an appropriate amount of RE elements can improve the magnetic properties of non-oriented silicon steel at lower annealing temperatures, which indicates a great potential to produce high-grade non-oriented silicon steel by a lower carbon emission production process.

### 4.2. Effects of Multiple Metallurgical Parameters on Iron Loss

Total iron loss can be separated into three components: hysteresis loss (*P*_h_), eddy current loss (*P*_e_), and abnormal loss (*P*_a_) [49,50]. The iron loss components are related to metallurgical parameters including grain size, texture, and inclusions [50,51,52]. Several models have been developed based on the texture parameter (*A*) [53], magnetic crystalline anisotropy parameter (*ε*) [54], and grain size [55,56]. However, there remains a deficiency—grain size, texture, and inclusions are not simultaneously incorporated into an iron loss model.

#### 4.2.1. Hysteresis Loss

Magneto-crystalline anisotropy energy density (*E*) is used to quantitatively characterize the texture of non-oriented silicon steel:

(1) $$E=K_{1}\left(\cos^{2}\alpha_{1}\cos^{2}\alpha_{2}+\cos^{2}\alpha_{2}\cos^{2}\alpha_{3}+\cos^{2}\alpha_{1}\cos^{2}\alpha_{3}\right)+K_{2}\cos^{2}\alpha_{1}\cos^{2}\alpha_{2}\cos^{2}\alpha_{3}$$

where *K*_1_ = (47.7–21.2 at%Si-3.8 at%Al) kg/m^−3^, *K*_1_ = 5 kg/m^−3^, (cos *α*_1_, cos *α*_2_, cos *α*_3_) is the unit vector of rolling direction expressed in crystal coordinates.

It was found that there is a linear dependence of *E* on *A*, even though *E* and *A* were derived from different calculation procedures. Additionally, a linear relationship between *E* and *P*_h_ was also observed. The average magneto-crystalline anisotropy energy density ($\bar{E}$) in polycrystalline samples can be expressed as follows:

(2) $$\bar{E}=\overset{N}{\underset{i=1}{\sum }}V\left(g_{i}\right)E\left(g_{i}\right)\;$$

where *V*(*g_i_*) represents the volume fraction of grains with orientation *i*, and *E*(*g_i_*) denotes the magneto-crystalline anisotropy energy density of grains with orientation *i*.

Dijkstra et al. [57] proposed that inclusions affect the energy of magnetic domain walls in relation to the particle size. De Campos et al. [58] suggested a dependence of coercivity (*H*_c_) on the volume fraction of inclusions:

(3) $$\Delta H_{c}=\frac{a}{12}\frac{\mu_{0}B_{s}^{2}}{B_{m}}V_{\mathrm{Inclusion}}\left\{\begin{array}{c} a=0.50\;if\;d>d_{0}\\ a=0.75\;if\;d\approx d_{0}\\ a=0\;\;\;\;\;\;\;if\;d<d_{0} \end{array}\right.$$

where *d* is inclusion particle size, *d*_0_ (0.1~0.2 μm) is the thickness of the magnetic domain wall, and *V* is the volume fraction of inclusions.

In this paper, a comprehensive model for *P*_h_ is proposed, which takes into account the characteristics of inclusions, grain size, and texture based on the aforementioned models:

(4) $$P_{h}=\left(c_{1}H_{c}\left(V_{\mathrm{Inclusion}},d\right)+c_{2}/D+c_{3}\bar{E}+c_{4}\right)B_{m}^{q}f$$

where *c*_1_, *c*_2_, and *c*_3_ represent the effects of inclusion, average grain size, and average magnetic crystalline anisotropy energy density, and *c*_4_ is a constant. The values of *c*_1_, *c*_2_, *c*_3_, and *c*_4_ are derived as 2.61 × 10^−3^, 4.71 × 10^−7^, 2.8 × 10^3^, 7.9 × 10^−3^ in the present study, respectively.

#### 4.2.2. Abnormal Loss

According to the Pry–Bean model, a larger grain size produces a greater abnormal loss [47]. The magnetic domain width (2*L*) and grain size satisfy 2*L*∝*D*^1/2^; an empirical formula for abnormal loss can be derived [52]:

(5) $$P_{a}=c_{3}t^{2}\sqrt{D}B_{m}^{2}f^{3/2}/\rho$$

In addition, for an identical grain size, the beneficial texture component, such as λ-fiber, has a coarser magnetic domain in contrast to detrimental components, such as γ-fiber. However, the aforementioned models for abnormal loss do not account for texture. Here, the influence of texture on abnormal loss is incorporated by the volume fraction of λ-fiber:

(6) $$P_{a}=\left(c_{5}\sqrt{D}+c_{6}V_{001}\right)t^{2}B_{m}^{2}f^{3/2}/\rho$$

where *c*_5_ and *c*_6_ represent the effects of average grain size and beneficial texture on the *P*_a_. *c*_5_ and *c*_6_ are derived as 0.28 and 0.15 in the present study, respectively.

**Figure 18:**
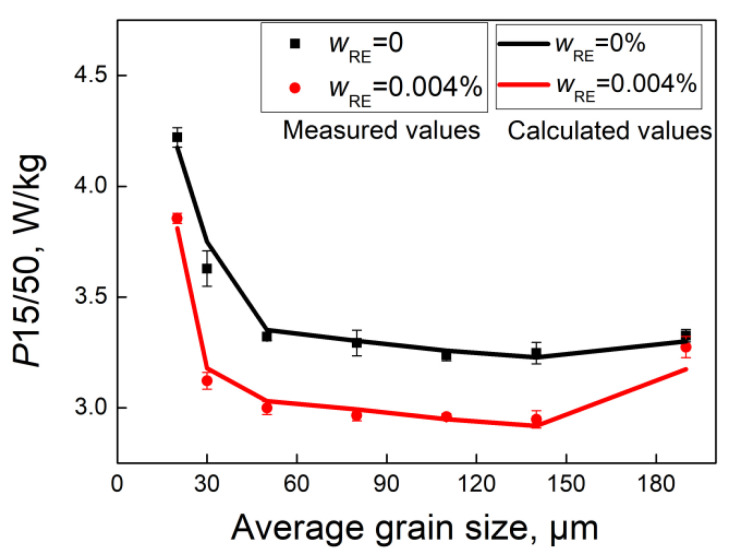
The measured and calculated iron loss values for different RE elements with average grain size of silicon steel sheets annealed at 800~1100 °C for 5 min.

Based on the proposed model, the iron loss associated with varying *E*, grain size, and inclusion obtained from the samples with different mixed RE elements can be calculated. Figure 18 compares the measured and calculated loss values of silicon steels without RE elements and with a mixed RE element of 0.004%.

The correlation coefficient (*R*^2^) of 0.95 confirms the validity of the proposed model. Iron loss initially decreases and then increases as grain size increases, with the optimal value occurring at a grain size of 100–150 μm. Trace addition of mixed RE elements results in a significant reduction in iron loss by effectively coarsening inclusions and improving recrystallization texture. The iron loss calculation model can provide guidance for the design of high-grade non-orientated silicon steel based on the multiple effects of inclusions, grain size, and texture.

## 5. Conclusions

Effects of mixed rare earth (RE) elements on the microstructure, texture, inclusion, and magnetic properties of non-oriented silicon steel were systematically investigated. The main conclusions are as follows:

1. The RE-rich inclusion particles preferentially form and adsorb other fine particles smaller than 1 μm to transform into spherical or ellipsoidal composites in the case of trace addition RE elements (0.004–0.030%). Consequently, the larger grain size can be obtained at lower annealing temperatures. However, a higher content of RE elements (0.072%) leads to finer and more stable RE-rich particles.
2. Recrystallization texture is improved in terms of increased λ fiber and reduced γ fiber by trace RE elements (0.004–0.030%) due to the advantages in grain size and quantity of λ fiber during nucleation, which results from the grain boundary segregation of RE elements and the heterogeneity in microstructure and texture of cold-rolled sheets. In contrast, a higher content of RE elements (0.072%) leads to strong recrystallization γ fiber.
3. The enhanced magnetic properties of higher B_50_ and lower P_15/50_ are achieved at lower annealing temperature ranges through trace addition of RE elements (0.004%). The trace mixed RE elements exhibit a great potential to enhance magnetic properties in a lower carbon emission approach by multiple effects on the microstructure, texture, and inclusion in non-oriented silicon steel.
4. An iron loss calculation model that incorporates multiple factors including grain size, texture, and inclusions is proposed. The iron loss calculation model can provide guidance for the design of high-grade non-orientated silicon steel based on the multiple effects of inclusions, grain size, and texture.

## Figures and Tables

**Table 1:** Chemical compositions of silicon steels (wt.%).

No.	C	Si	Al	Mn	S	P	Ce	Nd	La	Pr	Fe
1	0.003	3.14	1.06	0.27	0.001	0.009	0	0	0	0	Bal.
2	0.004	3.12	1.15	0.26	0.001	0.009	0.001	0.001	0.001	0.001	Bal.
3	0.004	3.10	1.17	0.26	0.001	0.009	0.013	0.007	0.008	0.002	Bal.
4	0.004	3.11	1.15	0.27	0.001	0.008	0.031	0.016	0.018	0.007	Bal.

## Data Availability

The original contributions presented in the study are included in the article/Supplementary Material, further inquiries can be directed to the corresponding authors.

## Supplementary Materials

The following supporting information can be downloaded at: https://www.mdpi.com/article/10.3390/ma18020401/s1, Figure S1. Average grain size through thickness in just-completed recrystallized silicon steel sheets.
