# Supplementary material for: The Multiple Effects of RE Element Addition in Non-Oriented Silicon Steel
Source: Materials (Basel). 2025 Jan 16;18(2):401. doi: 10.3390/ma18020401 (PMC11766840; doi:10.3390/ma18020401)
Supplement: Supplementary file 1 [file materials-18-00401-s001.zip › materials-3359054-supplementary.pdf]

The Multiple Effects of RE Element Addition in Non-Oriented Silicon Steel

Guobao Li [1,*], Yongjie Yang [1], Zhenghua He [2,3] and Yuhui Sha [2,*]

[1] Baoshan Iron & Steel Cooperation Limited, Shanghai 201999, China; yangyongjie@baosteel.com
[2] Key Laboratory for Anisotropy and Texture of Materials (Ministry of Education), Northeastern University, Shenyang 110819, China; hezhenghua@mail.neu.edu.cn or hezhh@sut.edu.cn
[3] School of Materials Science and Engineering, Shenyang University of Technology, Shenyang 110870, China
* Correspondence: ligb@baosteel.com (G.L.); yhsha@mail.neu.edu.cn (Y.S.); Tel.: +86-24-83691569 (Y.S.)

Figure S1. Average grain size through thickness in just completed recrystallized silicon steel sheets.

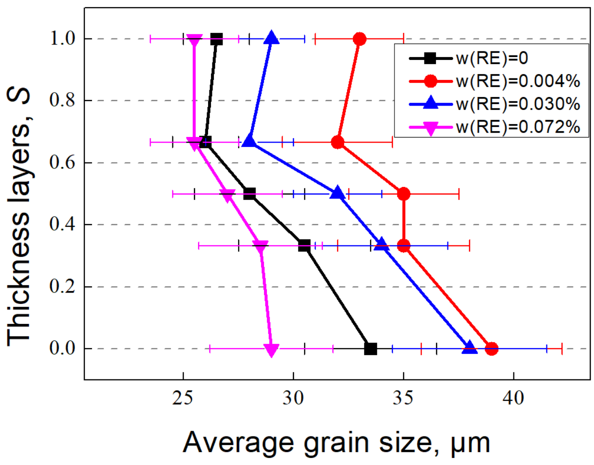

Figure S1. Average grain size through thickness in just completed recrystallized silicon steel sheets.
